# Spontaneous rupture of renal cell carcinoma: A case report from Mogadishu Somali Turkey hospital training and research hospital

**DOI:** 10.1016/j.ijscr.2025.111190

**Published:** 2025-03-24

**Authors:** Ubah Mumin Ali Osman, Yahye Sheikh Abdulle Hassan, Hanan Hassan Hirei

**Affiliations:** a Department of Emergency Medicine, Mogadishu Somali Turkey Hospital Training and Research Hospital, Somalia; b Faculty of Medicine and Health Sciences, Jamhuriya University of Science and Technology, Somalia; c Department of Radiology, Mogadishu Somali Turkey Hospital Training and Research Hospital, Somalia

**Keywords:** Renal cell carcinoma, Wunderlich's syndrome, Flank pain, Nephrectomy

## Abstract

**Introduction:**

Spontaneous rupture of renal masses, known as Wunderlich's syndrome, is a rare but potentially life-threatening condition characterized by acute perirenal hemorrhage. Renal cell carcinoma (RCC) is a less common cause of these ruptures, which may serve as the initial manifestation of the disease. This case report highlights the diagnostic and management challenges of spontaneous RCC rupture.

**Case presentation:**

A 40-year-old female presented with acute right-sided abdominal pain for 4 h. She exhibited tachycardia (120 bpm). Bedside ultrasonography suggested a renal mass, confirmed by contrast-enhanced computed tomography (CECT), which revealed a right renal mass with a large retroperitoneal hematoma. Rapid hemoglobin decline and severe pain raised suspicion of renal rupture. Emergency laparotomy with nephrectomy was performed, and histopathology confirmed clear cell renal cell carcinoma. The patient recovered uneventfully.

**Clinical discussion:**

Spontaneous rupture of renal cell carcinoma (RCC) is rare and presents diagnostic and therapeutic challenges. Contrast-enhanced computed tomography (CEST) is the most reliable imaging modality for confirming diagnoses. Differential diagnoses include vascular anomalies, coagulopathies, and angiomyolipomas. Surgical resection is the primary treatment, and prompt intervention is vital to reduce complications and improve prognosis. Postoperative surveillance ensures complete tumor removal and monitors for recurrence.

**Conclusion:**

This case highlights the rarity of spontaneous RCC rupture and its diagnostic challenges. CEST is crucial for diagnosis, with the differential diagnoses including vascular disorders, coagulopathies, and renal tumor. Surgical resection remains the mainstay of treatment, with timely intervention essential for improving patient outcomes.

## Highlights

• Rarity: Spontaneous rupture of renal cell carcinoma is a rare, life-threatening condition and can be the initial manifestation of the disease.
• Diagnostic Challenges: Acute flank pain and hemodynamic instability resemble other conditions, requiring high clinical suspicion and advanced imaging for accurate diagnosis.
• Imaging Importance: Contrast-enhanced CT is critical for diagnosing ruptured RCC, and identifying the mass, hematoma, and adjacent structural involvement.
• Surgical Intervention: Emergency nephrectomy is essential for stabilizing the patient and managing ruptured RCC effectively.
• Clinical Awareness: RCC rupture should be considered in acute flank pain cases, even without risk factors, to ensure timely diagnosis and intervention.

## 1 Introduction

Abdominal pain is a frequent complaint in emergency departments, accounting for a substantial proportion of patient visits [1]. Wunderlich's syndrome is a rare, potentially life-threatening condition resulting from spontaneous rupture of a renal mass [2]. This condition, while uncommon, requires prompt recognition and intervention.

Spontaneous perirenal hemorrhage, the hallmark of Wunderlich's syndrome, has diverse etiologies including benign and malignant neoplasms, vascular abnormalities, coagulopathies, and infections [3,4]. Angiomyolipomas (AML) are the most frequent neoplastic cause [5], but RCC, an aggressive malignancy of the kidney, represents a rarer etiology [6]. Spontaneous rupture can be the initial sign of the RCC, making timely diagnosis challenging and crucial [7].

The mechanism of spontaneous rupture in RCC is complex, possibly involving direct tumor invasion of capsular or vascular structures, elevated venous pressure due to renal vein thrombosis, or tumor necrosis leading to fragility [7,8]. Clinically, patients may present with sudden onset flank pain, often accompanied by hematuria and a palpable abdominal mass [9].

CESR is the gold-standard imaging modality for diagnosing and planning the management of renal ruptures [10,11]. In regions with limited healthcare resources, such as Somalia, delayed diagnosis and treatment may increase the risk of complications, emphasizing the need for awareness and timely intervention [12]. This case report aims to contribute to the limited literature on spontaneous RCC rupture by presenting a unique clinical scenario, discussing diagnostic challenges, and highlighting critical management strategies. This work aligns with the SCARE 2023 criteria [13].

## 2 Case presentation

A 40-year-old female arrived at the emergency department with a 4-hour history of acute, sharp abdominal pain, predominantly localized to the right side. On physical examination, the patient exhibited tenderness in the right flank, and her vital signs revealed tachycardia (120 bpm), while other vital signs were within normal limits. The patient had no prior chronic illness, trauma, or known kidney disease. Her past medical history was unremarkable, with no known risk factors for RCC, such as smoking or a family history of renal malignancies.

Bedside ultrasonography identified the right renal mass, prompting further evaluation. Laboratory tests showed a hemoglobin level of 11.2 g/dL, dropping to 8.2 g/dL, prompting fluid resuscitation and blood transfusion. Coagulation parameters were within normal limits.

**Fig. 1:**
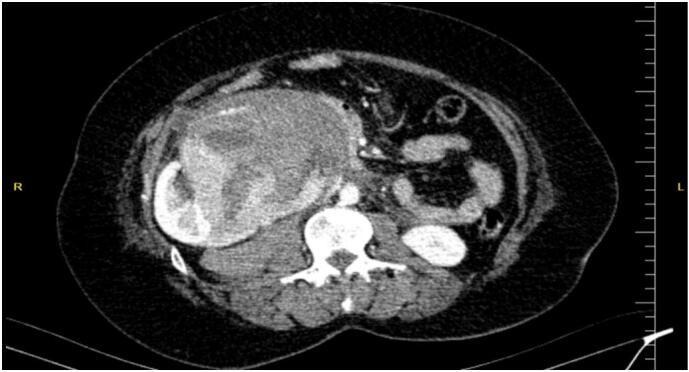
A Heterogenous enhancing mass arising in the hilum of the right kidney with perirenal, retroperitoneal, and pelvic hematoma.

**Fig. 2:**
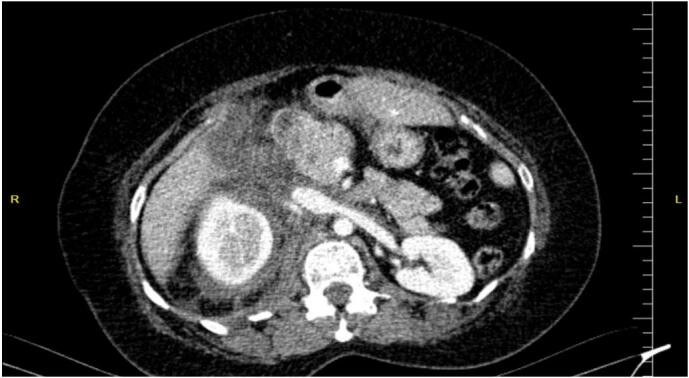
Displacement of the pancreatic head and duodenum superiorly and to the left.

Due to the disproportionate severity of the pain relative to clinical findings, contrast-enhanced computed tomography (CT) of the abdomen was performed. The scan revealed a heterogeneously enhancing mass arising from the hilum of the right kidney, compressing the inferior vena cava and adjacent structures, including the pancreatic head and duodenum. A large perirenal, retroperitoneal, and pelvic hematoma measuring 24 × 60 × 81 mm was detected (Fig. 1, Fig. 2).

**Fig. 3:**
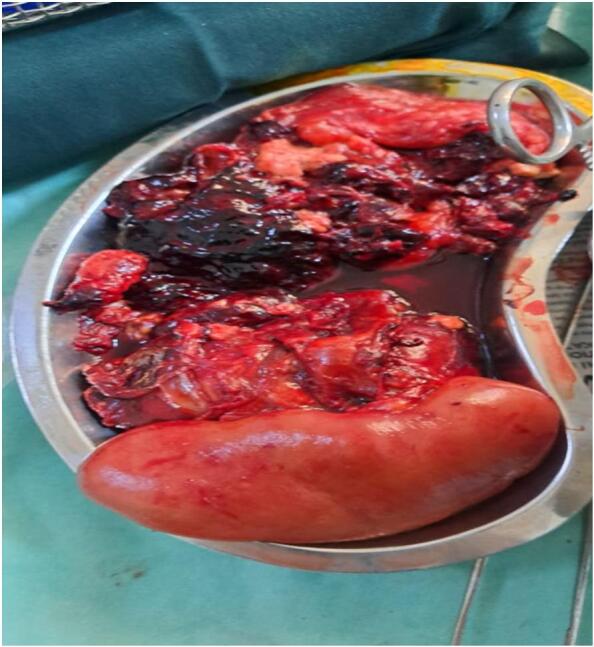
Total right kidney excision and a large hematoma.

The urology team performed an urgent laparotomy with a right nephrectomy and hematoma evacuation (Fig. 3). Histological examination confirmed clear cell eosinophilic renal cell carcinoma.

Following surgery, the patient was monitored in the intensive care unit for three days. Upon stabilization, she was transferred to the hospital's inpatient service. No postoperative complications were noted, and the patient was discharged with scheduled for outpatient follow-up appointments.

## 3 Discussion

This case presents a rare presentation of a spontaneous ruptured clear cell renal cell carcinoma (RCC) emphasizing its diagnostic and therapeutic challenges. While the classic triad of hematuria, flank pain, and palpable abdominal mass is commonly associated with RCC, rupture can be the initial manifestation, complicating timely diagnosis [14].

The patient's clinical course, marked by severe pain disproportionate to initial findings, rapid hemodynamic decline requiring blood transfusion, and the presence of a large retroperitoneal hematoma, is consistent with reports of spontaneous renal hemorrhage secondary to underlying renal tumors [15].

CECT was crucial for diagnosis, identifying the heterogeneously enhancing mass at the renal hilum and its involvement with adjacent structures. This imaging modality remains the most reliable and sensitive tool for detecting renal neoplasms, assessing hemorrhage extent, and guiding surgical planning [11,16] Alternative imaging options, such as MRI, can be considered when CECT is contraindicated.

Previous studies have documented similar cases of spontaneous RCC rupture, further supporting the role of imaging in timely diagnosis and intervention [17]. However, our case is distinguished by its acute presentation and the extent of hemorrhage, necessitating immediate nephrectomy.

As shown in Table 1, spontaneous rupture of RCC has been documented in patients with diverse demographic and clinical presentations. The majority of cases involved middle-aged to elderly males, with predisposing factors such as end-stage renal disease or hypertension. In contrast, our case was a comparatively young female with no known risk factors, rendering this case unique. Additionally, while some cases were managed through embolization, most required surgical intervention, underscoring the importance of prompt surgical management in hemodynamically unstable patients.

**Table 1:** Summary of reported cases of spontaneous renal rupture secondary to renal cell carcinoma.

Author(s), year	Patient age, sex	Presenting symptoms	Pre-existing conditions/risk factors	Imaging findings	Tumor type (histology)	Treatment	Outcome
Present case	40, female	Acute right flank pain	None reported	Right renal mass with perirenal, retroperitoneal, and pelvic hematoma compressing adjacent structures	Clear cell eosinophilic RCC	Urgent laparotomy with right nephrectomy and hematoma evacuation	Discharged with outpatient follow-up
Durak et al., 2014	79, male	Found dead at home	None reported	Right kidney mass with rupture, retroperitoneal hematoma	Renal cell carcinoma	Autopsy	Death due to internal bleeding
Kim et al., 2011 (Case 1)	47, male	Acute left flank pain during hemodialysis	ESRD, Hemodialysis	Ruptured enhancing renal mass, retroperitoneal hematoma; 3.2 × 2.5 cm mass	Clear cell type RCC, Fuhrman grade 1 (pT1a)	Open radical nephrectomy	Discharged without complications
Kim et al., 2011 (Case 2)	49, male	Acute left flank pain during hemodialysis	ESRD, Hemodialysis, Acquired cystic kidney disease	Hyperdense hematoma, no solid tumor initially identified; Multiple cysts in both kidneys; 10 × 7.2 × 13 cm hematoma	Clear cell type RCC, Fuhrman grade 1 (pT1a)	Open nephrectomy	Discharged without complications
Chauhan et al., 2020	46, male	Severe right lower abdominal pain	Non-smoker, No other medical comorbidities	Heterogeneous mass in lower pole of right kidney, perinephric hemorrhage and retroperitoneal hematoma; 8.0 × 7.5 × 7.0 cm exophytic lesion	Clear cell renal cell carcinoma grade 2	Emergency laparotomy and right nephrectomy	Uneventful post-operative period
Grubb et al., 2017	22, male	Sudden onset left lower quadrant and flank pain	Hypertension (well controlled)	Fragmented left kidney with retroperitoneal and intraperitoneal hemorrhage; Congenital duplication of the left kidney and a 3.1 cm lobulated, cystic mass on the cortex of the duplicated left kidney	Cystic mass identified; Exact RCC Type Not Specified	Angiography and coil embolization, followed by observation; congenital kidney duplication identified later	Discharged after 3 days
Petrut et al., 2020	69, male	Imagistic suspicion of both left renal and left adrenal tumoral masses	Previous laparoscopic right radical nephrectomy and adrenalectomy for clear cell renal carcinoma (48 months prior); Right inguinal hernia repair and cholecystectomy with perforated duodenal ulcer and hyperthyroidism	3 tumoral formations on the anterior valve of the left renal parenchyma (21/22 mm, 17/12 mm, 15/14 mm near the renal sinus). 3 hypervascularized conglomerated nodular masses (27 mm) on the caudal extremity of the left adrenal gland	Metachronous metastases as multi-focal tumors on a left solitary kidney and a left adrenal tumor of the contralateral CCRCC (previously treated)	3D laparoscopic transperitoneal left cytoreductive nephrectomy and left adrenalectomy (23 min warm ischemia)	Discharged on the 6th day in good general status. No post-operative hemodialysis. No signs of disease recurrence or metastases at the present date (last CT scan in June 2020)

The differential diagnosis of RCC is broad and includes vascular disorders, coagulopathies, infections, and renal tumors [18]. However, neoplastic causes are among the most common and particularly important to exclude. Among neoplasms, angiomyolipomas (AML) and RCC are primary concerns. However, in contrast to the reported case that described a spontaneously ruptured AML, our patient's imaging findings suggested RCC, which was subsequently confirmed by histopathological examination [19].

The optimal management of ruptured RCC involves initial stabilization followed by surgical resection [5]. Our patient underwent an emergency right radical nephrectomy, which is the treatment of choice for localized RCC with suspected tumor spillage due to rupture [20]. Surgical precision and efficiency are essential in RCC management, where complete resection is crucial, even when confronted with technical challenges such as metachronous RCC metastases in a solitary kidney, or tumor spillage due to rupture [21].

Following nephrectomy, postoperative surveillance, including periodic imaging and laboratory evaluation, is critical to detect recurrence. Red flag signs, such as unexplained weight loss, persistent pain, or hematuria, should prompt immediate reassessment.

## 4 Conclusion

This case underscores the need for high clinical suspicion of RCC rupture in patients with acute flank pain, even without known risk factors. Early recognition and prompt surgical intervention are critical for favorable outcomes. CEST remains the gold standard for diagnosis, and histopathology is essential for confirmation. Postoperative surveillance is necessary to ensure complete tumor removal and monitor for recurrence.

## Author contribution

Ubah Mumin Ali Osman: Conceptualized and wrote the manuscript, performed literature review, and contributed to patient management. Hanan Hassan Hirei: Assisted in patient care, collected case data, and revised the manuscript. Yahye Sheikh Abdulle Hassan: Designed the case report, provided final approval of the manuscript, and acted as the corresponding author.

## Consent

Written informed consent was obtained from the patient for publication and any accompanying images. A copy of the written consent form is available for review by the editor-in-chief of this journal upon request.

## Ethical approval

According to the regulations of the review board of the Mogadishu Somali Turkish Training and Research Hospital, institutional review board approval is not required for case reports.

## Guarantor

Dr. Ubah Mumin Ali Osman.

## Research registration number

N/A.

## Funding

The authors of this study declare that there are no funding sources.

## Conflict of interest statement

The authors declare no conflicts of interest related to this case report.
